# Dynamic face processing impairments are associated with cognitive and positive psychotic symptoms across psychiatric disorders

**DOI:** 10.1038/s41537-021-00166-z

**Published:** 2021-08-10

**Authors:** Hayley Darke, Suresh Sundram, Simon J. Cropper, Olivia Carter

**Affiliations:** 1 Melbourne School of Psychological Sciences, University of Melbourne, Melbourne, VIC Australia; 2 The Florey Institute of Neuroscience and Mental Health, Parkville, VIC Australia; 3 Department of Psychiatry, School of Clinical Sciences, Monash University, Melbourne, VIC Australia; 4 Mental Health Program, Monash Health, Melbourne, VIC Australia

**Keywords:** Biomarkers, Human behaviour

## Abstract

Impairments in social cognition—including recognition of facial expressions—are increasingly recognised as a core deficit in schizophrenia. It remains unclear whether other aspects of face processing (such as identity recognition) are also impaired, and whether such deficits can be attributed to more general cognitive difficulties. Moreover, while the majority of past studies have used picture-based tasks to assess face recognition, literature suggests that video-based tasks elicit different neural activations and have greater ecological validity. This study aimed to characterise face processing using video-based stimuli in psychiatric inpatients with and without psychosis. Symptom correlates of face processing impairments were also examined. Eighty-six psychiatric inpatients and twenty healthy controls completed a series of tasks using video-based stimuli. These included two emotion recognition tasks, two non-emotional facial identity recognition tasks, and a non-face control task. Symptoms were assessed using the Positive and Negative Syndrome Scale. Schizophrenia and bipolar disorder groups were significantly impaired on the emotion-processing tasks and the non-face task compared to healthy controls and patients without psychosis. Patients with other forms of psychosis performed intermediately. Groups did not differ in non-emotional face processing. Positive symptoms of psychosis correlated directly with both emotion-processing performance and non-face discrimination across patients. We found that identity processing performance was inversely associated with cognition-related symptoms only. Findings suggest that deficits in emotion-processing reflect symptom pathology independent of diagnosis. Emotion-processing deficits in schizophrenia may be better accounted for by task-relevant factors—such as attention—that are not specific to emotion processing.

## Introduction

Social cognition is increasingly recognised as a core deficit in schizophrenia^1^. One component of social cognition is the ability to extract emotional cues from faces. Impaired emotion recognition is associated with poorer social and occupational functioning in schizophrenia, and may also mediate the relationship between social functioning and broader neurocognitive deficits^2–4^. Furthermore, deficits in emotion recognition appear to precede the onset of psychosis, and may predict the conversion to schizophrenia in clinically high-risk populations^5^.

The exact mechanics that underlie impairments in emotion-processing are an ongoing source of debate. There is consensus, however, that individuals with schizophrenia are significantly impaired in their ability to recognise emotions compared to healthy controls and other psychiatric disorders^6,7^. Meta-analyses estimate these effect sizes to be quite large, e.g., *d* = −0.91*, d* = −0.85, and *g* = 0.89 respectively^6,8,9^. It remains unclear whether these reported emotion-processing deficits are indeed specific to facial expressions, or whether they could be due to impairments in processing structural aspects of faces in general, or processing visual stimuli more generally. Notably, the ability to recognise the identity of a face may also be impaired in schizophrenia, although previous studies have produced varying results^10,11^. As facial emotion-processing and identity-processing are believed to be underlain by largely separate neural routes^12,13^, the comparison of these abilities allows us to better characterise these impairments and their relevant correlates.

The N170 is an event-related potential thought to relate to the structural encoding of faces and is increased when viewing faces compared to other complex objects. In patients with schizophrenia, the N170 is attenuated for both faces and other complex objects, although findings vary across studies (see paper by Salisbury et al., 2019^14^). Overall, this suggests a generalised visual processing impairment that is not specific to faces, but may contribute to impairments on face-processing tasks.

Research in healthy populations suggests that using dynamic stimuli—rather than static images—to investigate emotion-processing confers a range of advantages, including increased ecological validity and greater accuracy^15^. Despite this, only a handful of studies have employed dynamic face stimuli to investigate emotion-processing in schizophrenia. Deficits in recognising dynamic face-specific emotions have been reported by several studies of schizophrenia patients^16–19^. Notably, static and dynamic tasks may tap into different patterns of difficulties. In one study, performance on dynamic tasks correlated with greater positive symptoms, while performance on static tasks correlated with negative symptoms^17^. In another study, patients’ performance on a dynamic emotion-recognition task correlated with IQ and other cognitive measures, while performance in healthy controls correlated with a face memory task and a social cognition task only. These results concur with research using static stimuli (e.g., Bediou et al., 2007^20^) and lend weight to the argument that emotion-processing deficits in schizophrenia may be accounted for by cognitive deficits.

A further question is whether the face processing deficits observed in schizophrenia are shared by other psychiatric disorders. There is some evidence for impaired face processing in bipolar disorder, particularly for recognising emotion, although the degree and persistence of these deficits remain contentious^21^. Patients with bipolar disorder have shown impaired performance compared to healthy controls in correctly labelling facial emotions^22–24^ as well as discriminating between different expressions^25–27^. Other studies have shown that patients with bipolar disorder require greater intensity to recognise emotions compared to controls^28,29^. In contrast, other studies report intact emotion recognition in bipolar disorder^30–32^ or in certain patient subsets, such as euthymic patients^22^.

Studies indicate some degree of impairment in recognising facial emotion in major depressive disorder (MDD), although the extent of these deficits remains unclear. Several reviews suggest that, on balance, there are mild but significant impairments in emotion-processing in MDD^33–35^. In particular, MDD patients tend to show a bias towards perceiving neutral or ambiguous expressions as sadness, and show a reduced ability to recognise all basic emotions except for sadness^36^. In contrast, a number of experimental studies have reported *no* difference in emotion-processing ability between patients with MDD and healthy controls^29,30,37–42^. This discrepancy is possibly due to a lack of power in these studies, as a recent meta-analysis suggests that the overall effect size for these deficits in MDD is quite small (Hedges *g* = −0.16)^36^.

With respect to the anxiety disorders, one meta-analysis examined emotion-processing impairments across 40 studies^43^. A large weighted mean effect size was found for post-traumatic stress disorder (Cohen’s *d* = −1.60), indicating substantial deficits in emotion recognition. However, only small or negligible effects were found for social phobia (*d* = 0.12), obsessive-compulsive disorder (*d* = −0.16), panic disorder (*d* = −0.25) and generalised anxiety disorder (*d* = −0.12). Whether these deficits indicate specific impairments in recognising emotion, or simply reflect more general difficulties with attentional control that typically accompany anxiety disorders^44^, remains to be established.

Face processing deficits may accompany specific symptoms which are found across disorders. For instance, emotion-processing deficits—albeit using *static* stimuli—have been predominantly associated with negative symptoms in schizophrenia, and to a lesser degree positive symptoms, later age of illness onset and inpatient status^6,45,46^. In contrast, the relationship between identity recognition impairments and symptoms has not been widely studied, and evidence is mixed. Two studies have reported a negative correlation between identity recognition performance in schizophrenia and both positive and negative symptoms^47,48^ while several report correlations with negative symptoms only^26,45,49–51^. To date, no studies have assessed symptom correlates with identity processing using dynamic stimuli.

**Fig. 1:**
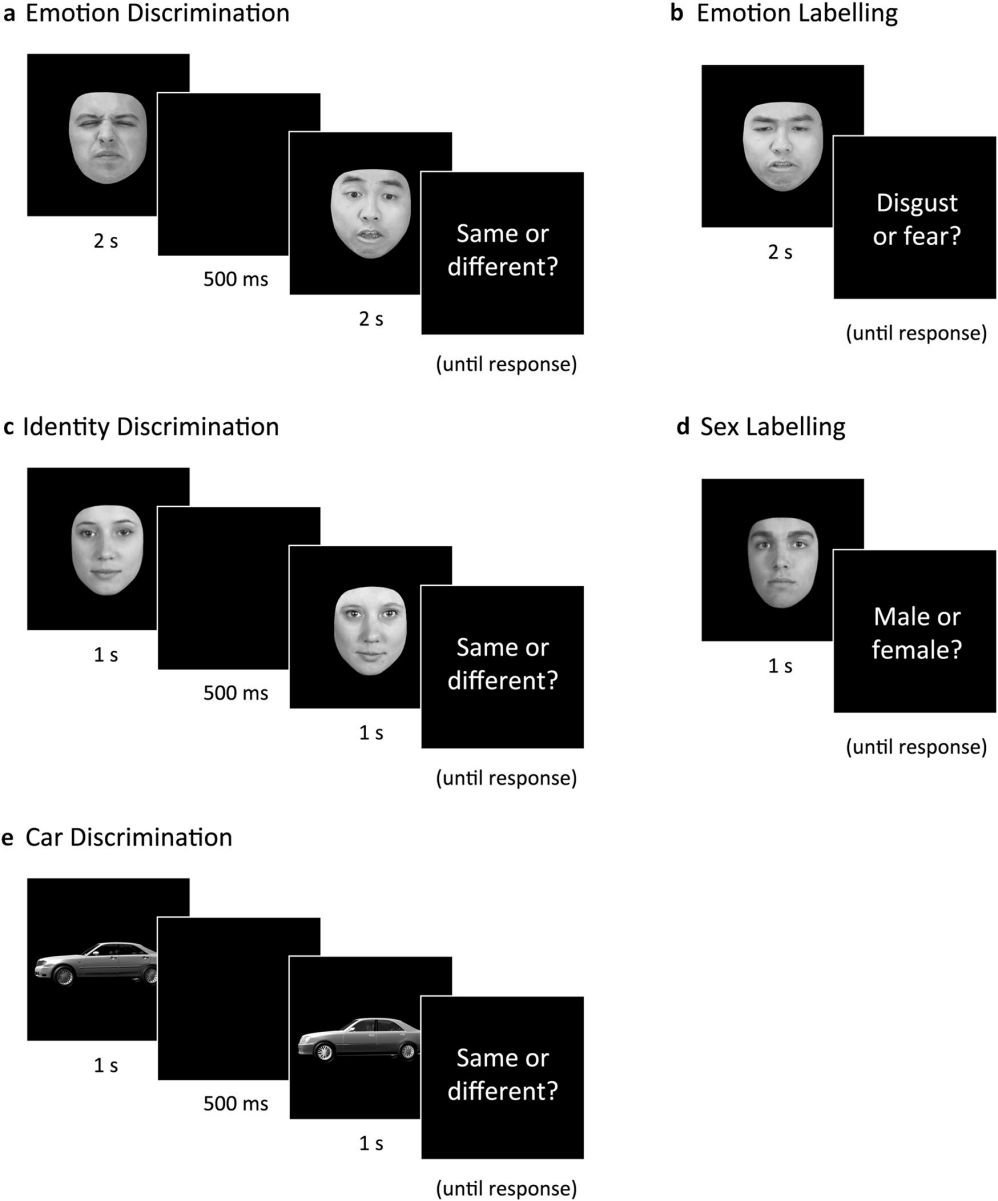
Example trials for each task illustrate a snapshot image of the dynamic video stimulus presented to participants. Participants were asked to report whether the feature of interest was the same or different across the two videos (**a**, **c**, and **e**) or to label the emotion illustrated as disgust/fear (**b**) or the sex was male/female (**d**). Morphing of the video files was used to create different levels of task difficulty. Further details regarding the stimuli set and morphing procedure are provided in supplemental Figures 1–3 and the methods paper by Darke and colleagues^52^.

The current study aimed to address these issues by assessing participants with a range of psychiatric disorders (*n* = 86) and healthy controls (*n* = 20) using four different emotion and identity processing tasks (see Fig. 1). To ascertain whether these deficits generalise beyond face processing, performance was also compared on an equivalent task using non-face stimuli. Novel dynamic video-based stimuli were developed for use in all tasks (see Darke et al., 2019^52^ for further details). The aims were (a) to explore whether impairments in emotion-processing can be explained by more general deficits in non-emotional face processing or non-face processing, (b) to assess whether face processing impairments are shared by other psychiatric disorders, and (c) to investigate symptom correlates of face processing ability across psychiatric disorders.

## Results

### Demographics

Demographics and symptom ratings are shown in Table 1. Pearson’s chi-square test revealed that the sex makeup of groups did not differ significantly as a function of diagnosis, *X*^*2*^(4) = 6.18, *p* = 0.186. One-way ANOVAs were performed with Group as a between-subjects factor and Age, Years of Education, and estimated FSIQ as within-subjects factors. A significant effect was found for Years of Education, *F*(4,101) = 2.50, *p* = 0.048, *ŋ*_p_^2^ = 0.09. Post-hoc *t* tests (Bonferroni corrected) revealed a significant difference of 2.11 years between the Control and Schizophrenia group (*p* = 0.02). FSIQ estimates were also found to differ between groups, *F*(4,93) = 2.52, *p* = 0.047, *ŋ*_p_^2^ = 0.10. Post-hoc *t* tests (Bonferroni corrected) revealed a significant difference of 8.38 points between the Control and Schizophrenia spectrum groups (*p* = 0.03). Age did not differ significantly between groups, *F*(4,101) = 0.75, *p* = 0.56, *ŋ*_p_^2^ = 0.03.

**Table 1:** Mean participant demographics and questionnaire scores by group.

	Schizophrenia spectrum *n* = 36	BPAD *n* = 15	Other psychotic disorders *n* = 17	Non-psychotic disorders *n* = 18	Healthy controls *n* = 20
Age (years)	34.44 (9.44) range 19–53	36.60 (14.80) range 19–65	30.65 (7.61) range 18–43	36.17 (13.57) range 19–59	34.05 (10.72) range 18–56
Males/females	23/13	9/6	12/5	6/12	12/8
(% male)	(65%)	(60%)	(71%)	(33%)	(60%)
Education (years)	10.89 (2.55)*	11.40 (1.76)	11.65 (3.28)	11.83 (2.48)	13.00 (1.59)*
Premorbid IQ^a^	100.33 (10.93)*	103.62 (8.83)	102.65 (9.94)	105.69 (10.73)	108.70 (6.57)*
Illness duration (years)	9.12 (7.56)	8.87 (11.07)	4.15 (6.31)	8.45 (9.97)	–
Antipsychotic daily dose^b^	367.2 mg (276.2)	358.9 mg (52.6)	225.9 mg (115.6)	155.6 mg (212.4)	–
Benzodiazepine daily dose^c^	35.00 mg (26.30)	44.00 mg (35.25)	–	23.13 mg (17.72)	–
*PANSS*					
Positive scale	17.28 (5.45)	18.47 (4.17)	14.24 (4.35)	8.56 (1.76)^d^	
Negative scale	11.53 (3.72)	9.13 (1.89)	9.94 (3.46)	12.28 (4.85)	
General psychopathology	31.53 (6.47)	31.60 (4.98)	31.53 (7.05)	31.67 (4.51)	

Note: SD in parentheses.

*Bonferroni-corrected *t* tests revealed a significant group difference at *p* < 0.05.

^a^Due to dyslexia, illiteracy, or poor English proficiency, IQ estimates were not available for four patients in the schizophrenia spectrum group, two in the other group, and two in the non-psychosis group.

^b^Chlorpromazine equivalent dose.

^c^Diazepam equivalent dose.

^d^Positive symptom scores for the non-psychosis group were significantly lower than all other groups (*p*s = 0.002 to <0.001).

One-way ANOVAs conducted with the four inpatient groups only revealed no significant group differences in mean duration of illness, *F*(3,82) = 1.42, *p* = 0.25, *ŋ*_p_^2^ = 0.05, mean daily dose of antipsychotics, *F*(3,61) = 1.68, *p* = 0.18, *ŋ*_p_^2^ = 0.08, or daily benzodiazepine dose, *F*(2,13) = 0.63, *p* = 0.55, *ŋ*_p_^2^ = 0.09. Medication status for each group is shown in Supplementary Table 1.

### PANSS subscales

One-way ANOVAs were run with group as IV (excluding healthy controls), and Positive, Negative, and General Psychopathology scores as DVs. A significant main effect was found for Positive Symptoms, *F*(3,82) = 18.76, *p* < 0.001, *ŋ*_p_^2^ = 0.41. Bonferroni corrected post hoc tests revealed, not surprisingly, that the Non-psychosis group had significantly lower Positive symptom scores than all other groups (*p* = 0.002 to <0.001). The Other group trended towards having significantly lower Positive symptom scores compared with the bipolar group (*p* = 0.055). No other group differences approached significance.

### Task performance in healthy controls

A repeated-measures ANOVA was conducted to determine whether difficulty varied across the five dynamic tasks in healthy controls. Briefly, accuracy for the Identity Discrimination task was significantly higher compared to the Sex Labelling and Emotion Discrimination tasks, however performance across all other tasks was of a comparable level. Within-group comparisons for the four patient groups can be found in the supplemental appendix. A main effect of task was found for each of the five within-group analyses (*p* < 0.001).

### Group differences

Repeated–measures ANOVAs were conducted on raw accuracy data across morphing levels for each of the five groups (see supplementary appendix for analyses). As all groups showed the same pattern of performance, data for each task was collapsed across morphing levels for subsequent analyses.

A 5 ×5 MANOVA revealed a significant main effect for group, *F*(4,99) = 2.93, *p* < 0.001, *ŋ*_p_^2^ = 0.13 (Pillai’s Trace). Univariate tests revealed significant effects of group for all tasks except Sex Labelling.

**Fig. 2:**
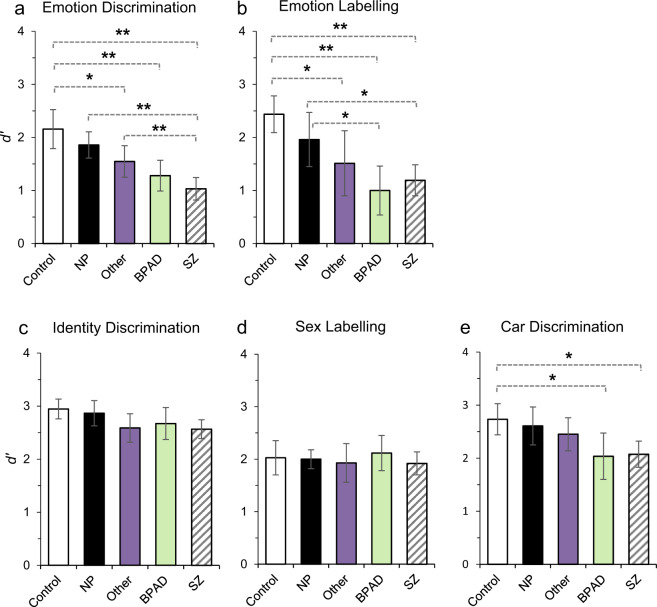
Mean *d*’ performance for the schizophrenia spectrum (SZ), BPAD, other psychosis (Other), non-psychosis (NP) and control groups across five tasks. A higher *d*’ value indicates better performance. Significant differences between groups are indicated with dotted lines, **p < 0.01; *p < 0.05. Error bars represent 95% confidence intervals.

Emotion discrimination (Fig. 2a) showed a univariate effect of group (*F*(4,99) = 14.18, *p* < 0.001, *ŋ*_p_^2^ = 0.36). The Schizophrenia group performed significantly poorer than all other groups (Healthy control: *p* < 0.001, Hedges’ *g* = 1.77; Non-psychosis: *p* < 0.001, Hedges’ *g* = 1.58; Other psychosis: *p* = 0.02, Hedges’ *g* = 0.97) except Bipolar disorder (*p* > 0.999, Hedges’ *g* = 0.52). The healthy control group also significantly outperformed the other psychosis (*p* = 0.03, Hedges’ *g* = 0.88) and Bipolar disorder (*p* < 0.001, Hedges’ *g* = 1.28) groups. The non-psychosis group trended towards significantly outperforming the Bipolar group (*p* = 0.07, Hedges’ *g* = 1.14) but did not differ significantly from healthy controls (*p* > 0.999, Hedges’ *g* = 0.45) or the Other psychosis group (*p* > 0.999, Hedges’ *g* = 0.58).

A similar pattern of results were found for Emotion Labelling (Fig. 2b; Univariate effect of group: *F*(4,99) = 9.19, *p* < 0.001, *ŋ*_p_^2^ = 0.27). The healthy control group outperformed the Schizophrenia (*p* < 0.001, Hedges’ *g* = 1.77), Bipolar (*p* < 0.001, Hedges’ *g* = 1.85) and Other psychosis groups (*p* = 0.02, Hedges’ *g* = 0.95). The Non-psychosis group outperformed the Schizophrenia (*p* = 0.02, Hedges’ *g* = 0.97) and Bipolar (*p* = 0.03, Hedges’ *g* = 1.02) groups, but did not differ significantly from the healthy control (*p* > 0.999, Hedges’ *g* = 0.54) or Other psychosis groups (*p* > 0.999, Hedges’ *g* = 0.40).

Identity discrimination (Fig. 2c) had a main effect of group, (*F*(4,99) = 2.47, *p* = 0.049, *ŋ*_p_^2^ = 0.09). There was a trend for the Schizophrenia group to perform more poorly than healthy controls (*p* = 0.09, Hedges’ *g* = 0.76), however pairwise comparisons revealed no other significant differences between any groups (*p* values = 0.33–0.99, Hedge’s *g*s = 0.03–0.79).

No effect of group was found for Sex Labelling, (*F*(4,99) = 0.363, *p* = 0.84, *ŋ*_p_^2^ = 0.01). An effect of morphing level was seen, with lower accuracy for 40% male/60% female faces (See Supplementary Fig. 9E), however overall performance was consistent across all groups (see Fig. 2d).

Unexpectedly, a significant univariate effect of group was also found for Car Discrimination (Fig. 2e; *F*(4,99) = 4.31, *p* = 0.003, *ŋ*_p_^2^ = 0.15). Pairwise comparisons revealed that this was driven by the Control group significantly outperforming the Schizophrenia (*p* = 0.01, Hedges’ *g* = 0.99) and Bipolar groups (*p* = 0.03, Hedges’ *g* = 1.0). No other comparisons approached significance (*p*s = 0.12–0.99, Hedges’ *g*s = 0.10–0.75).

### Response bias—c

Mean values of *c* ranged from 0.02 to 0.88 across groups and tasks. Multivariate ANOVA revealed no significant main effect of group, *F*(4,99) = 0.84, *p* = 0.67, *ŋ*_p_^2^ = 0.04. Pairwise comparisons showed no differences between groups on any task, suggesting that response bias did not differ between groups, and is therefore unlikely to account for group differences in task performance.

### Age, education and FSIQ

Pearson correlations were conducted to determine if age, years of education, or FSIQ estimates predicted performance on any of the tasks. It was found that increasing age correlated with worsening *d*’ for Emotion Labelling (*r* = −0.197, *p* = 0.04), but not other tasks. FSIQ estimates produced no significant correlations. Years of education correlated positively with performance on Emotion Discrimination (*r* = 0.260, *p* = 0.007) only. To determine if years of education could account for group differences on the Emotion Discrimination task, analyses were re-run excluding participants with fewer than 11 years of education (eliminating 13 patients in the schizophrenia group, 5 Bipolar disorder, 6 Other and 6 Non-psychosis). One-way ANOVA revealed a significant mean effect of group *F*(4,70) = 10.64, *p* < 0.001, *ŋ*_p_^2^ = 0.38. Post-hoc tests showed that the schizophrenia group performed significantly lower than the control (*p* < 0.001), Non-psychosis (*p* = 0.001) and Other groups (*p* = 0.02). The control group also outperformed the Bipolar group (*p* = 0.01). Thus, limited years of education was unlikely to account for the group differences shown on these tasks.

### Illness duration and medication

Pearson correlations revealed that illness duration correlated negatively with task performance on Emotion Discrimination (*r* = −0.337, *p* = 0.002), Emotion Labelling (*r* = −0.224, *p* = 0.04) and Car Discrimination (*r* = −0.298, *p* = 0.005), and trended negatively with Identity Discrimination (*r* = −0.204, *p* = 0.06). Hierarchical regression analyses revealed that, after controlling for Age, Illness Duration continued to significantly predict performance on Emotion Discrimination (change in *R*^2^ = 0.09, *F*(1,83) = 8.37, *p* = 0.005) and Car Discrimination (change in *R*^*2*^ = 0.13, *F*(1,83) = 12.27, *p* = 0.001), but no longer predicted performance on Emotion Labelling (change in *R*^*2*^ = 0.01, *F*(1,83) = 1.27, *p* = 0.26). This suggests that patients with a longer illness duration performed more poorly on all tasks except for Sex Labelling, regardless of group, however this cannot account for the group differences observed.

In recognition of the potential sedating effects of benzodiazepines^53^ and antipsychotics^54^, correlations between medications and performance were examined. Benzodiazepine daily dose (*n* = 16) correlated negatively with performance on Emotion Labelling (*r* = −0.519, *p* = 0.04) and Car Discrimination (*r* = −0.535, *p* = 0.033) and trended towards significance for Identity Discrimination (*r* = −0.458, *p* = 0.07). Mean antipsychotic daily dose (*n* = 84) produced no significant correlations.

To determine if benzodiazepine use could account for the group differences in task performance, a MANOVA was run excluding the 17 inpatients who had taken benzodiazepines. As before, a significant main effect was found for group, *F*(4,83) = 2.50, *p* < 0.001, *ŋ*_p_^2^ = 0.13 (Pillai’s Trace) and univariate tests still revealed significant, or trending towards significant effects of group for four of the five tasks: Identity Discrimination (*F*(4,83) = 2.07, *p* = 0.09, *ŋ*_p_^2^ = 0.09), Emotion Discrimination (*F*(4,83) = 11.22, *p* < 0.001, *ŋ*_p_^2^ = 0.35), Emotion Labelling (*F*(4,83) = 7.01, *p* < 0.001, *ŋ*_p_^2^ = 0.25), and Car Discrimination (*F*(4,83) = 3.96, *p* = 0.005, *ŋ*_p_^2^ = 0.16). This suggests that although benzodiazepine use was related to poorer performance on some tasks, it cannot account for the group differences reported in this study.

### Correlations with clinical symptoms

Table 2 shows correlations between PANSS subscales and task performance. Positive Symptoms were negatively correlated with all tasks except Sex Labelling (*r*s = −0.31 to 0.41). General Psychopathology scores correlated negatively with Car Discrimination only (*r* = 0.37). No correlations with Negative Symptoms approached significance for any of the tasks.

**Table 2:** Pearson correlations between PANSS scores and d prime performance on the five dynamic tasks.

	Emotion discrimination	Emotion labelling	Identity discrimination	Sex labelling	Car discrimination
	*r* [95% CI]	*r* [95% CI]	*r* [95% CI]	*r* [95% CI]	*r* [95% CI]
Positive symptoms	−0.41* [−0.55, −0.24]	−0.33* [−0.50, −0.13]	−0.31* [−0.50, −0.08]	0.06 [−0.10, 0.22]	−0.35* [0.55, 0.11]
Negative symptoms	−0.06 [−0.33, 0.21]	−0.20 [−0.39, 0.03]	−0.21 [−0.41, 0.00]	−0.09 [−0.31, 0.13]	−0.24 [−0.48, 0.03]
General Psychopathology	−00.11 [−0.34, 0.14]	−0.16 [−0.35, 0.05]	−0.22 [−0.44, 0.05]	−0.12 [−0.33, 0.09]	−0.37* [−0.53, −0.17]

Note: *Indicates a significant correlation, where the 95% confidence interval for *r* does not include 0.

Spearman-rank correlations between individual items on the PANSS and task performance are shown in Table 3. When considered overall, it appears that classically positive symptoms—such as Delusions, Grandiosity, Suspiciousness, and Unusual Thought Content—correlated negatively with performance on the two Emotion tasks, and with Car Discrimination. In contrast, no positive symptoms correlated with the two Identity tasks (with the exception of Unusual Thought Content, which was positively correlated with Sex Labelling).

**Table 3:** Spearman rank correlations between individual PANSS items and d prime performance on the five dynamic tasks.

	Emotion discrimination	Emotion labelling	Identity discrimination	Sex labelling	Car discrimination
	*r*_*s*_ [95% CI]	*r*_*s*_ [95% CI]	*r*_*s*_ [95% CI]	*r*_*s*_ [95% CI]	*r*_*s*_ [95% CI]
Delusions	−0.42* [−0.60, −0.23]	−0.24* [−0.45, −0.01]	−0.18 [−0.39, 0.06]	0.09 [−0.14, 0.30]	−0.27* [−0.47, −0.07]
Conceptual disorganisation	−0.50* [−0.65, −0.32]	−0.29* [−0.49, −0.06]	−0.26* [−0.46, −0.03]	0.15 [−0.09, 0.37]	−0.34* [−0.55, −0.10]
Hallucinations	−0.08 [−0.27, 0.12]	−0.09 [−0.30, 0.13]	−0.15 [−0.36, 0.07]	−0.07 [−0.30, 0.17]	−0.12 [−0.32, 0.09]
Excitement	−0.16 [−0.38, 0.07]	−0.20 [−0.41, 0.03]	−0.22 [−0.42, 0.04]	0.11 [−0.13, 0.34]	−0.31* [−0.51, −0.06]
Grandiosity	−0.32* [−0.52, −0.09]	−0.29* [−0.49, 0.08]	−0.17 [−0.38, 0.07]	0.23* [0.02, 0.45]	−0.24* [−0.43, −0.02]
Suspiciousness	−0.28* [−0.48, −0.06]	−0.25* [−0.46, −0.03]	−0.14 [−0.36, 0.09]	0.02 [−0.20, 0.22]	−0.25* [−0.46, −0.02]
Hostility	−0.06 [−0.28, 0.15]	−0.10 [−0.30, 0.10]	−0.12 [−0.33, 0.12]	−0.06 [−0.28, 0.19]	−0.11 [−0.33, 0.11]
Blunted affect	0.02 [−0.20, 0.25]	−0.10 [−0.31, 0.12]	−0.20 [−0.40, 0.02]	−0.05 [−0.26, 0.17]	−0.14 [−0.36, 0.13]
Emotional withdrawal	0.00 [−0.27, 0.27]	−0.02 [−0.23, 0.20]	−0.03 [−0.22, 0.18]	0.02 [−0.14, 0.16]	0.001 [−0.17, 0.16]
Poor rapport	0.07 [−0.16, 0.30]	0.04 [−0.17, 0.23]	−0.03 [−0.24, 0.19]	−0.08 [−0.27, 0.12]	−0.05 [−0.29, 0.19]
Passive apathetic withdrawal	0.21 [−0.03, 0.43]	0.09 [−0.12, 0.30]	0.11 [−0.09, 0.30]	−0.06 [−0.28, 0.15]	0.12 [−0.08, 0.31]
Difficulty in abstract thinking	−0.24* [−0.44, −0.04]	−0.31* [−0.47, 0.13]	−0.32* [−0.50, −0.12]	−0.04 [−0.26, 0.18]	−0.26* [−0.45, −0.05]
Lack of spontaneity	0.05 [−0.18, 0.30]	0.06 [-0.18, 0.27]	-0.03 [-0.24, 0.18]	-0.22 [-0.40, 0.002]	-0.13 [-0.36, 0.09]
Stereotyped thinking	−0.29* [−0.49, −0.06]	−0.34* [−0.51, −0.12]	−0.29* [−0.47, −0.07]	0.03 [−0.21, 0.27]	−0.28* [−0.47, −0.04]
Somatic concern	0.17 [−0.07, 0.36]	0.17 [−0.04, 0.37]	0.18 [−0.05, 0.40]	0.01 [−0.23, 0.23]	0.01 [−0.22, 0.20]
Anxiety	0.18 [−0.02, 0.38]	0.18 [−0.03, 0.38]	0.04 [−0.18, 0.24]	−0.09 [−0.31, 0.13]	0.03 [−0.20, 0.26]
Guilt feelings	0.22* [0.03, 0.40]	0.12 [−0.09, 0.31]	0.05 [−0.16, 0.27]	−0.10 [−0.31, 0.012]	0.18 [−0.004, 0.39]
Tension	0.004 [−0.21, 0.22]	0.05 [−0.17, 0.26]	−0.01 [−0.24, 0.24]	0.004 [−0.24, 0.026]	−0.16 [−0.038, 0.07]
Mannerisms and posturing	0.002 [−0.20, 0.22]	0.04 [−0.19, 0.27]	0.07 [−0.12, 0.25]	0.04 [−0.19, 0.28]	−0.04 [−0.020, 0.13]
Depression	0.34* [0.15, 0.52]	0.13 [−0.08, 0.32]	0.10 [−0.12, 0.29]	−0.13 [−0.35, 0.009]	0.11 [−0.012, 0.034]
Motor retardation	0.09 [−0.13, 0.29]	−0.02 [−0.24, 0.17]	−0.03 [−0.21, 0.15]	−0.16 [−0.34, 0.004]	−0.11 [−0.29, 0.12]
Uncooperativeness	−0.17 [−0.36, 0.04]	−0.10 [−0.30, 0.11]	−0.10 [−0.34, 0.16]	−0.14 [−0.35, 0.10]	−0.17 [−0.40, 0.05]
Unusual thought content	−0.46* [−0.65, −0.23]	−0.33* [−0.52, −0.12]	−0.15 [−0.38, 0.09]	0.28* [0.06, 0.48]	−0.27* [−0.48, −0.02]
Disorientation	0.02 [−0.16, 0.20]	−0.12 [−0.36, 0.13]	−0.26* [−0.41, −0.08]	−0.17 [−0.38, 0.007]	−0.24* [−0.42, −0.03]
Poor attention	−0.28* [−0.47, −0.07]	−0.29* [−0.47, −0.06]	−0.39* [−0.57, −0.014]	−0.23 [−0.44, 0.004]	−0.49* [−0.63, 0.29]
Lack of judgement and insight	−0.39* [−0.57, −0.18]	−0.40* [−0.57, −0.19]	−0.19 [−0.40, 0.004]	0.04 [−0.20, 0.28]	−0.25* [−0.48, −0.01]
Disturbance of volition	−0.02 [−0.22, 0.18]	−0.08 [−0.24, 0.07]	−0.29* [−0.45, −0.010]	−0.16 [−0.39, 0.12]	−0.14 [−0.32, 0.06]
Poor impulse control	−0.07 [−0.27, 0.15]	−0.08 [−0.28, 0.13]	−0.11 [−0.31, 0.013]	−0.11 [−0.36, 0.15]	−0.17 [−0.39, 0.05]
Preoccupation	−0.14 [−0.33, 0.07]	−0.06 [−0.28, 0.17]	−0.14 [−0.35, 0.007]	0.09 [−0.12, 0.031]	−0.30* [−0.47, −0.10]
Active social avoidance	−0.15 [−0.34, 0.06]	−0.14 [−0.36, 0.07]	−0.14 [−0.36, 0.010]	−0.03 [−0.24, 0.018]	−0.22 [−0.44, 0.02]

Note: *Indicates a significant correlation, where the 95% confidence interval for *r*_*s*_ does not include 0.

The other trend to note is that performance on all tasks (except Sex Labelling) tended to correlate negatively with *cognitive* symptoms, such as Conceptual Disorganisation, Difficulty in Abstract Thinking, Stereotyped Thinking, Poor Attention, Disorientation, and Lack of Judgement and Insight. These items also appear to drive the association reported between General Psychopathology subscale scores and Car Discrimination performance. Interestingly, almost no correlations were found with affective symptoms such as Depression and Blunted Affect. The only exception was that higher Depression and Guilt Feelings was associated with *better* performance on Emotion Discrimination, but not other tasks.

## Discussion

The aim of the current study was to determine the diagnostic specificity and symptom correlates of impairments in facial emotion and identity processing using dynamic tasks. Results revealed that groups with bipolar disorder, schizophrenia, and ‘other’ (non-schizophrenia) psychotic disorders were significantly impaired on the emotion tasks compared to healthy controls, while patients with non-psychotic disorders were unimpaired. In contrast, all patient groups showed relatively intact performance on the two identity processing tasks. Unexpectedly, patients with schizophrenia and bipolar disorder also showed deficits on a non-face comparison task. Analysis of symptom correlates suggested that tasks of facial emotion, identity, and non-face discrimination were associated with different patterns of symptoms.

These results revealed significant emotion-processing deficits in patients with bipolar I disorder which were comparable to patients with schizophrenia. These findings are consistent with previous studies of patients with bipolar disorder using static stimuli, which typically report emotion-processing deficits regardless of task design^22–27^. Unexpectedly, the current study showed no significant difference in performance between the bipolar and schizophrenia groups. This is contrary to previous research^26,31,34,55–57^ although several other studies have also produced a null result^30,41,58^. This finding may reflect a lack of power due to the small number (*n* = 16) of bipolar patients in this sample. Alternatively, this sample may have been more impaired due to the exclusion of bipolar II disorder (non-psychotic) patients from this group, a subtype that in some cases has been shown to be less impaired in emotion-processing compared to bipolar I patients^22,59^.

This study demonstrates an impairment in dynamic emotion-processing in non-schizophrenia psychoses. This finding supports the idea that non-schizophrenia forms of psychosis produce similar deficits to those seen in schizophrenia, and likely involve similar neural mechanisms^60^. Moreover, the finding that emotion recognition was unimpaired in patients with *non*-psychotic disorders is consistent with studies reporting broadly intact performance in major depression^29,39,41,42^, certain anxiety disorders^43^, and borderline personality disorder^61^. Note that, although some meta-analyses suggest that these disorders are associated with mild deficits in emotion recognition (particularly misinterpretation of threatening faces) these are typically of a much smaller effect size than those seen in schizophrenia, and it is possible that the current study lacked the power to detect subtler deficits^36,43^. Future work should aim to replicate these results in larger studies to ensure the findings are robust and representative of the broader population with these disorders.

Our results suggest that, irrespective of diagnosis, patients with positive symptoms such as delusions, suspiciousness, and unusual beliefs are most likely to show emotion-processing difficulties compared to those with other symptoms. This result is at odds with the majority of past research using static stimuli, which predominantly report associations with *negative* symptoms^30,51,62–66^ However, this finding is consistent with that of Johnston and colleagues (2010) who found that positive symptoms correlated with dynamic emotion-processing, while negative symptoms correlated with static emotion-processing. Given that dynamic faces elicit different patterns of brain activation compared to static faces^67^, it is possible that positive and negative symptoms may have differential effects on these brain networks. It is worth noting, however, that subsequent studies using dynamic emotion tasks have not replicated this dissociation between positive and negative symptoms^16,18^.

Interestingly, the current study found that performance on two dynamic identity-processing tasks was not associated with positive symptoms *or* negative symptoms. The use of our dynamic stimuli that were designed specifically to examine identity processing in patients, provides important new evidence about the specificity of perceptual alternations seen across this psychiatric population.

The current study revealed no significant impairments in identity processing in any of the groups examined. This finding is consistent with previous studies in bipolar disorder^23,27,68^, major depression^37^, and some studies of schizophrenia^20,37,69^. Unexpectedly, it was found that the schizophrenia and bipolar groups were both significantly impaired on a non-face discrimination task compared to healthy controls. It is possible that the schizophrenia group showed preserved identity processing because the processing of non-emotional face information is mediated by rapid, largely automatic perceptual processes^70^. In contrast, discrimination of less familiar stimuli, such as cars, may require a greater level of cognitive effort or attentional control, and may therefore be more sensitive to subtler cognitive impairment.

The observation that cognitive-related symptoms correlated negatively with task performance suggests that patients with more generalised cognitive difficulties tended to be less accurate overall, regardless of stimulus type. This finding is consistent with previous studies indicating associations between cognitive factors and emotion-processing in schizophrenia^64,71,72^. This finding is not unexpected, particularly given the predominance of attentional difficulties in psychiatric disorders such as schizophrenia^73^. However, it does highlight the pervasive impact of generalised cognitive deficits, even on tasks that are intended to tap into specialised areas of perceptual deficit, such as emotion-processing. The current results align with the view that emotion-processing deficits in schizophrenia may be accounted for by more general task-relevant factors, such as attentional disturbance^74^. It is important to note, however, that our results do not rule out the presence of an overlapping emotion-specific deficit. Future studies would benefit from including standardised tests of cognition, particularly attention and visual working memory, to better control for these factors and clarify the relationship between aspects of cognitive impairment and emotion-processing ability.

In conclusion, using dynamic stimuli this study found that patients with schizophrenia and bipolar disorder showed similar deficits in emotion-processing, and non-face discrimination compared to healthy controls and patients with non-psychotic illnesses. Examination of symptoms across disorders indicated that positive symptoms of psychosis correlated with both emotion-processing performance and non-face discrimination across patients. Uniquely, we found that identity processing performance was associated with cognitive-related symptoms only. Findings align with the views that emotion-processing deficits may be accounted for by more general task-relevant factors, such as attentional disturbance seen in psychotic disorders, regardless of diagnostic category.

## Method

### Participants

Eighty-six inpatients were recruited from an acute psychiatry unit in Melbourne, Australia. All participants were inpatients at the time of participation. Final diagnoses (DSM-IV criteria) were obtained from discharge reports provided by the treating psychiatrist and verified by the research psychiatrist (Sundram). Patients were categorised into four groups: 36 schizophrenia-spectrum (including schizophrenia, schizoaffective disorder, and first episode psychosis), 15 bipolar disorder (bipolar-affective disorder with a history of psychotic symptoms), 17 “other” psychotic disorders (including drug induced-psychosis, major depression with psychosis, borderline personality disorder [PD] with hallucinations, and schizotypal PD), and 18 non-psychotic disorders (including bipolar II disorder, major depression, generalised anxiety disorder, borderline PD, and situational crisis – none of whom had ever experienced symptoms of psychosis).

Twenty non-clinical controls were recruited via online advertising. All were free from neurological injury, psychiatric illness or substance use disorder by self-report, and were not taking psychoactive medication. All participants received monetary compensation for their time and gave written informed consent. Inpatient participant consent was signed in the presence of an impartial witness. This study was approved by Melbourne Health and University of Melbourne Human Research Ethics Committees.

### Demographics

Participants completed the National Adult Reading Test (NART)^75^, and a demographics questionnaire. Patients additionally reported current medication, illness duration, and were assessed using the Positive and Negative Syndrome Scale (PANSS)^76^. PANSS interviews were conducted by a graduate student with extensive experience and all ratings were reviewed by an experienced PANSS rater and psychiatrist (Sundram).

### Emotion discrimination

Detailed explanation of task development can be found in Darke and colleagues^52^ and are available for download from http://go.unimelb.edu.au/e3t6. Stimuli used were 2000 ms videos of faces changing from neutral expressions to either disgust or fear, adapted from the MMI-Facial Expression Database^77,78^ and the Facial Expressions and Emotion Database (FEED)^79^. Fear and disgust were chosen because these expressions are not easily confused with one another in healthy controls (unlike anger and disgust, or fear and surprise^80^) and, unlike positive expressions, are more likely to elicit emotion-recognition impairments in clinical populations, such as in schizophrenia^81^ and bipolar disorder^21^.

Faces were edited to remove non-face cues (e.g., hair or glasses) and were presented centrally in greyscale against a black background. Stimuli were 5x4cm viewed at a distance of approximately 50 cm (5.7×4.6° of visual angle). To vary the intensity of emotion, peak expression frames from each video were “morphed” together with neutral frames to create new stimuli using Fantamorph 5^82^. Original videos consisted of six unique individuals (3 male, 3 female) each showing one expression of disgust and one of fear. These 12 videos were morphed to create five levels of expression intensity (33%, 50%, 67%, 83%, and 100%), totalling 60 stimuli (examples shown in Supplementary Figs. 1–3). In each trial one expression was shown, then followed by a second face of a different individual showing either the same or different expression (Fig. 1a). Pairs of expressions were always shown at the same intensity level. Participants were instructed to state aloud whether each pair of faces showed the “same” or “different” emotion.

### Emotion labelling

Stimuli used were the same as those in Emotion Discrimination. Each expression was shown for 2000 ms. Half of trials were “disgust” and half were “fear”. Participants were instructed to state aloud whether each face more closely resembled “fear” or “disgust”.

### Identity discrimination

Stimuli used were videos of faces showing non-emotive facial movements, such as opening the mouth or raising the eyebrows. Animations were created using the same methods described for Emotion Discrimination, except that video of different individuals (of the same sex) were morphed together to vary the degree of similarity between faces. Six pairs of unique individuals (3 male, 3 female) were used. Each pair was morphed to create six new animations ranging from one identity to the other at 20% increments, totalling 36 stimuli.

In each trial, a “pure” face (either 0% or 100%) was shown, followed by a second face from the same set that was either 0%, 20%, 40%, 60%, 80%, or 100% different. Participants verbally responded whether each pair of stimuli were “same” or “different”.

### Sex labelling

Stimuli used were identical to Identity Discrimination above, with the exception that each identity was morphed with an opposite-sex identity instead of a same-sex identity. Six sets of 6 face animations were created, ranging from male to female. Half of the trials were “male” (i.e.,: 60%, 80%, or 100% male) and half were “female” (0%, 20%, and 40% male). Participants were instructed to state aloud whether each face more closely resembled “male” or “female”.

### Car discrimination

Stimuli used were 1000 ms videos of 3D car models rotating from a side view to a 45° view. 3D meshes were obtained online via a free 3D modelling website^83^ then edited and animated using 3Ds Max Design^84^. Twelve unique cars were animated and paired with similar looking models. For each trial, one car video was shown for 1000 ms, followed by a 500 ms blank screen, then a second car video. Participants verbally responded whether each pair of stimuli were “same” or “different”.

### General procedure

Participants completed the five computerised tasks in one of four counterbalanced orders. Prior to each task, participants completed practice trials with feedback. For the three Discrimination tasks, participants were instructed to say whether each pair of stimuli (either faces, cars or emotions) were the same or different (the ratio of same/different trials was 50:50). Identity Discrimination and Car Discrimination consisted of 120 trials each (see Fig. 1a, c, and e). As Emotion Discrimination required longer presentation times, this task was reduced to 100 trials to limit participant fatigue.

For the two Labelling tasks, participants were instructed to state whether each face more closely resembled “fear” or “disgust” (Emotion), and “male” or “female” (Sex), respectively (Fig. 1b and d). Sex Recognition consisted of 72 trials. After piloting, Emotion Recognition was reduced to 60 trials to reduce testing time. The experimenter logged all verbal responses using a keyboard to reduce any impact of impulsive or impaired motor response mapping.

Testing took ~2 h to complete, and participants were permitted as many breaks as desired. Computerised tasks were completed on a laptop computer (60 Hz, 16 inch screen size) at a comfortable viewing distance in a quiet distraction-free environment.

Results were analysed using the software package SPSS version 20. To limit response bias, percentage correct was converted to d’ scores using formulae recommended by MacMillan and Creelman^85^. For Sex Labelling and Emotion Recognition this was calculated as: d’ = z(Hit rate) – z(False alarms). For the three Discrimination tasks this value was then converted to a modified d’^85^. To avoid dividing by zero, Hit Rate and False Alarms were adjusted according to Corwin^86^. A measure of response bias, c, was also calculated using the formula: c = −0.5 [z(Hit rate)+z(False alarms)]^85^. Task performance was compared across the five groups using Repeated-Measures ANOVA and MANOVA (where the assumption of sphericity was violated). All post-hoc comparisons were Bonferroni-corrected to control for multiple comparisons. For Pearson and Spearman correlation, bootstrapping was used to calculate bias-corrected and accelerated (BCa) confidence intervals using 1000 resamples^87^.

### Reporting summary

Further information on research design is available in the Nature Research Reporting Summary linked to this article.

## Supplementary information

Reporting Summary

Supplementary Information

## Data Availability

The raw data that support the findings of this study are available from the corresponding author upon request.

## Code availability

The programmed tasks used in this study are available from the corresponding author upon request.

## References

[1] Green MF, Horan WP, Lee J (2015). Social cognition in schizophrenia. Nat. Rev. Neurosci..

[2] Irani F, Seligman S, Kamath V, Kohler C, Gur RC (2012). A meta-analysis of emotion perception and functional outcomes in schizophrenia. Schizophr. Res..

[3] Tsunoda T (2012). Altered face inversion effect and association between face N170 reduction and social dysfunction in patients with schizophrenia. Clin. Neurophysiol..

[4] Meyer MB, Kurtz MM (2009). Elementary neurocognitive function, facial affect recognition and social-skills in schizophrenia. Schizophr. Res.

[5] Corcoran CM (2015). Emotion recognition deficits as predictors of transition in individuals at clinical high risk for schizophrenia: a neurodevelopmental perspective. Psychol. Med..

[6] Kohler CG, Walker JB, Martin EA, Healey KM, Moberg PJ (2010). Facial emotion perception in schizophrenia: a meta-analytic review. Schizophr. Bull..

[7] Ventura J, Wood RC, Jimenez AM, Hellemann GS (2013). Neurocognition and symptoms identify links between facial recognition and emotion processing in schizophrenia: meta-analytic findings. Schizophr. Res..

[8] Chan RC, Li H, Cheung EF, Gong QY (2010). Impaired facial emotion perception in schizophrenia: a meta-analysis. Psychiatry Res..

[9] Savla GN, Vella L, Armstrong CC, Penn DL, Twamley EW (2013). Deficits in domains of social cognition in schizophrenia: a meta-analysis of the empirical evidence. Schizophr. Bull..

[10] Bortolon C, Capdevielle D, Raffard S (2015). Face recognition in schizophrenia disorder: a comprehensive review of behavioral, neuroimaging and neurophysiological studies. Neurosci. Biobehav. Rev..

[11] Darke H, Peterman JS, Park S, Sundram S, Carter O (2013). Are patients with schizophrenia impaired in processing non-emotional features of human faces?. Front Psychol..

[12] Haxby JV, Hoffman EA, Gobbini MI (2000). The distributed human neural system for face perception. Trends Cogn. Sci..

[13] Haxby, J. V., & Gobbini, M. I. In *The Oxford Handbook of Face Perception* (ed. M. H. Johnson & J. V. Haxby) 93–110 (Oxford University Press, 2011).

[14] Salisbury DF, Krompinger JW, Lynn SK, Onitsuka T, McCarley RW (2019). Neutral face and complex object neurophysiological processing deficits in long-term schizophrenia and in first hospitalized schizophrenia-spectrum individuals. Int. J. Psychophysiol..

[15] Dobs K, Bulthoff I, Schultz J (2018). Use and usefulness of dynamic face stimuli for face perception studies-a review of behavioral findings and methodology. Front Psychol..

[16] Behere RV, Venkatasubramanian G, Arasappa R, Reddy NN, Gangadhar BN (2011). First rank symptoms & facial emotion recognition deficits in antipsychotic naive schizophrenia: Implications for social threat perception model. Prog. Neuropsychopharmacol. Biol. Psychiatry.

[17] Johnston PJ (2010). Symptom correlates of static and dynamic facial affect processing in schizophrenia: evidence of a double dissociation?. Schizophr. Bull..

[18] Mendoza R (2011). Impairment of emotional expression recognition in schizophrenia: a Cuban familial association study. Psychiatry Res.

[19] Hargreaves A (2016). Detecting facial emotion recognition deficits in schizophrenia using dynamic stimuli of varying intensities. Neurosci. Lett..

[20] Bediou B (2007). Emotion recognition and genetic vulnerability to schizophrenia. Br. J. Psychiatry.

[21] Van Rheenen TE, Rossell SL (2013). Is the non-verbal behavioural emotion-processing profile of bipolar disorder impaired? A critical review. Acta Psychiatr. Scand..

[22] Lembke A, Ketter TA (2002). Impaired recognition of facial emotion in mania. Am. J. Psychiatry.

[23] Getz GE, Shear PK, Strakowski SM (2003). Facial affect recognition deficits in bipolar disorder. J. Int Neuropsychol. Soc..

[24] Van Rheenen TE, Rossell SL (2014). Let’s face it: facial emotion processing is impaired in bipolar disorder. J. Int Neuropsychol. Soc..

[25] Rossell SL (2013). Investigating affective prosody in psychosis: a study using the comprehensive affective testing system. Psychiatry Res..

[26] Addington J, Addington D (1998). Facial affect recognition and information processing in schizophrenia and bipolar disorder. Schizophr. Res.

[27] Bozikas VP, Tonia T, Fokas K, Karavatos A, Kosmidis MH (2006). Impaired emotion processing in remitted patients with bipolar disorder. J. Affect Disord..

[28] Brotman MA (2008). Risk for bipolar disorder is associated with face-processing deficits across emotions. J. Am. Acad. Child Adolesc. Psychiatry.

[29] Schaefer KL, Baumann J, Rich BA, Luckenbaugh DA, Zarate CA (2010). Perception of facial emotion in adults with bipolar or unipolar depression and controls. J. Psychiatr. Res..

[30] Edwards J, Pattison PE, Jackson HJ, Wales RJ (2001). Facial affect and affective prosody recognition in first-episode schizophrenia. Schizophr. Res..

[31] Vaskinn A (2007). The effect of gender on emotion perception in schizophrenia and bipolar disorder. Acta Psychiatr. Scand..

[32] Harmer CJ, Grayson L, Goodwin GM (2002). Enhanced recognition of disgust in bipolar illness. Biol. Psychiatry.

[33] Leppanen JM (2006). Emotional information processing in mood disorders: a review of behavioral and neuroimaging findings. Curr. Opin. Psychiatry.

[34] Kohler CG, Hoffman LJ, Eastman LB, Healey K, Moberg PJ (2011). Facial emotion perception in depression and bipolar disorder: a quantitative review. Psychiatry Res.

[35] Bourke C, Douglas K, Porter R (2010). Processing of facial emotion expression in major depression: a review. Aust. N. Z. J. Psychiatry.

[36] Dalili MN, Penton-Voak IS, Harmer CJ, Munafo MR (2015). Meta-analysis of emotion recognition deficits in major depressive disorder. Psychol. Med..

[37] Bediou B (2005). Facial expression and sex recognition in schizophrenia and depression. Can. J. Psychiatry.

[38] Gaebel W, Wolwer W (1992). Facial expression and emotional face recognition in schizophrenia and depression. Eur. Arch. Psychiatry Clin. Neurosci..

[39] Gollan JK, Pane HT, McCloskey MS, Coccaro EF (2008). Identifying differences in biased affective information processing in major depression. Psychiatry Res.

[40] Kan Y, Mimura M, Kamijima K, Kawamura M (2004). Recognition of emotion from moving facial and prosodic stimuli in depressed patients. J. Neurol. Neurosurg. Psychiatry.

[41] Derntl B, Seidel EM, Schneider F, Habel U (2012). How specific are emotional deficits? A comparison of empathic abilities in schizophrenia, bipolar and depressed patients. Schizophr. Res..

[42] Anderson IM (2011). State-dependent alteration in face emotion recognition in depression. Br. J. Psychiatry.

[43] Plana I, Lavoie MA, Battaglia M, Achim AM (2014). A meta-analysis and scoping review of social cognition performance in social phobia, posttraumatic stress disorder and other anxiety disorders. J. Anxiety Disord..

[44] Eysenck MW, Derakshan N (2011). New perspectives in attentional control theory. Personal. Individ. Differ..

[45] Martin F, Baudouin JY, Tiberghien G, Franck N (2005). Processing emotional expression and facial identity in schizophrenia. Psychiatry Res.

[46] van ‘t Wout M (2007). Exploring the nature of facial affect processing deficits in schizophrenia. Psychiatry Res..

[47] Chen Y, Norton D, McBain R, Ongur D, Heckers S (2009). Visual and cognitive processing of face information in schizophrenia: detection, discrimination and working memory. Schizophr. Res..

[48] Chen Y, Ekstrom T (2016). Perception of faces in schizophrenia: Subjective (self-report) vs. objective (psychophysics) assessments. J. Psychiatr. Res..

[49] Norton D, McBain R, Holt DJ, Ongur D, Chen Y (2009). Association of impaired facial affect recognition with basic facial and visual processing deficits in schizophrenia. Biol. Psychiatry.

[50] Chen Y, McBain R, Norton D (2015). Specific vulnerability of face perception to noise: a similar effect in schizophrenia patients and healthy individuals. Psychiatry Res..

[51] Baudouin JY, Martin F, Tiberghien G, Verlut I, Franck N (2002). Selective attention to facial emotion and identity in schizophrenia. Neuropsychologia.

[52] Darke H, Cropper SJ, Carter O (2019). A novel dynamic morphed stimuli set to assess sensitivity to identity and emotion attributes in faces. Front Psychol..

[53] Stewart SA (2005). The effects of benzodiazepines on cognition. J. Clin. Psychiatry.

[54] Kane JM, Sharif ZA (2008). Atypical antipsychotics: sedation versus efficacy. J. Clin. Psychiatry.

[55] Wynn JK, Jahshan C, Altshuler LL, Glahn DC, Green MF (2013). Event-related potential examination of facial affect processing in bipolar disorder and schizophrenia. Psychol. Med..

[56] Ruocco AC (2014). Emotion recognition deficits in schizophrenia-spectrum disorders and psychotic bipolar disorder: findings from the Bipolar-Schizophrenia Network on Intermediate Phenotypes (B-SNIP) study. Schizophr. Res..

[57] Goghari VM, Sponheim SR (2013). More pronounced deficits in facial emotion recognition for schizophrenia than bipolar disorder. Compr. Psychiatry.

[58] Bellack AS, Blanchard JJ, Mueser KT (1996). Cue availability and affect perception in schizophrenia. Schizophr. Bull..

[59] Derntl B, Seidel EM, Kryspin-Exner I, Hasmann A, Dobmeier M (2009). Facial emotion recognition in patients with bipolar I and bipolar II disorder. Br. J. Clin. Psychol..

[60] Paparelli A, Di Forti M, Morrison PD, Murray RM (2011). Drug-induced psychosis: how to avoid star gazing in schizophrenia research by looking at more obvious sources of light. Front Behav. Neurosci..

[61] Mitchell AE, Dickens GL, Picchioni MM (2014). Facial emotion processing in borderline personality disorder: a systematic review and meta-analysis. Neuropsychol. Rev..

[62] Fakra E, Jouve E, Guillaume F, Azorin JM, Blin O (2015). Relation between facial affect recognition and configural face processing in antipsychotic-free schizophrenia. Neuropsychology.

[63] Doop ML, Park S (2009). Facial expression and face orientation processing in schizophrenia. Psychiatry Res.

[64] Sachs G, Steger-Wuchse D, Kryspin-Exner I, Gur RC, Katschnig H (2004). Facial recognition deficits and cognition in schizophrenia. Schizophr. Res.

[65] Turetsky BI (2007). Facial emotion recognition in schizophrenia: when and why does it go awry?. Schizophr. Res.

[66] Gur RE (2006). Flat affect in schizophrenia: relation to emotion processing and neurocognitive measures. Schizophr. Bull..

[67] Arsalidou M, Morris D, Taylor MJ (2011). Converging evidence for the advantage of dynamic facial expressions. Brain Topogr..

[68] Venn HR (2004). Perception of facial expressions of emotion in bipolar disorder. Bipolar Disord..

[69] Chen Y, Cataldo A, Norton DJ, Ongur D (2012). Distinct facial processing in schizophrenia and schizoaffective disorders. Schizophr. Res..

[70] McKone, E. & Robbins, R. in *Oxford Handbook of Face Perception* (eds Andy Calder, Gillian Rhodes, Mark Johnson, & Jim Haxby) 149–176 (Oxford University Press, 2011).

[71] Silver H, Shlomo N, Turner T, Gur RC (2002). Perception of happy and sad facial expressions in chronic schizophrenia: evidence for two evaluative systems. Schizophr. Res..

[72] Bozikas VP (2006). Impaired perception of affective prosody in schizophrenia. J. Neuropsychiatry Clin. Neurosci..

[73] Keefe, R. S. & Harvey, P. D. Cognitive impairment in schizophrenia. *Handb. Exp. Pharmacol.*, 11-37, 10.1007/978-3-642-25758-2_2 (2012). 10.1007/978-3-642-25758-2_2 23027411

[74] Pomarol-Clotet E (2010). Facial emotion processing in schizophrenia: a non-specific neuropsychological deficit?. Psychol. Med.

[75] Crawford JR (1992). Estimation of premorbid intelligence in schizophrenia. Br. J. Psychiatry.

[76] Kay SR, Fiszbein A, Opler LA (1987). The positive and negative syndrome scale (PANSS) for schizophrenia. Schizophr. Bull..

[77] Pantic, M., Valstar, M., Rademaker, R. & Maat, L. In *2005 IEEE International Conference on Multimedia and Expo*. 5 pp.

[78] Valstar, M. & Pantic, M. In *Proc. 3rd Intern. Workshop on EMOTION (satellite of LREC): Corpora for Research on Emotion and Affect*. 65.

[79] Wallhoff, F., Schuller, B., Hawellek, M. & Rigoll, G. In *2006 IEEE International Conference on Multimedia and Expo*. 493-496.

[80] Jack RE, Garrod OGB, Schyns PG (2014). Dynamic facial expressions of emotion transmit an evolving hierarchy of signals over time. Curr. Biol..

[81] Grave, J., Soares, S. C., Martins, M. J. & Madeira, N. Facial emotion processing in schizophrenia: a review of behavioural and neural correlates. *International Journal of Clinical Neurosciences and Mental Health* **4**(Suppl. 3), S06 (2017).

[82] Abrosoft. *Fantamorph 5 [Computer software]*. *Retrieved from* http://www.fantamorph.com/. 2012).

[83] Studio ArchiDOM. 3D free model cars. Retrieved from http://www.archidom.net/ddd/mod_drive.htm. (2011).

[84] Autodesk Inc. *3DS Max design [Computer software]*. *Retrieved from* http://www.autodesk.com.au/products/3ds-max/overview, 2012).

[85] Macmillan, N. A. & Creelman, C. D. *Detection theory: a user’s guide*. (Cambridge University Press, 1991).

[86] Corwin J (1994). On measuring discrimination and response bias: unequal numbers of targets and distractors and two classes of distractors. Neuropsychology.

[87] DiCiccio TJ, Efron B (1996). Bootstrap confidence intervals. Stat. Sci..

