# Supplementary material for: Dynamic face processing impairments are associated with cognitive and positive psychotic symptoms across psychiatric disorders
Source: NPJ Schizophr. 2021 Aug 10;7:36. doi: 10.1038/s41537-021-00166-z (PMC8355323; doi:10.1038/s41537-021-00166-z)
Supplement: Supplementary file 1 — Reporting Summary [file 41537_2021_166_MOESM1_ESM.pdf]

nature research

Corresponding author(s): Olivia Carter

Last updated by author(s): Apr 19, 2021

Reporting Summary

Nature Research wishes to improve the reproducibility of the work that we publish. This form provides structure for consistency and transparency in reporting. For further information on Nature Research policies, see our Editorial Policies and the Editorial Policy Checklist.

Statistics

For all statistical analyses, confirm that the following items are present in the figure legend, table legend, main text, or Methods section.

n/a	Confirmed	
☐	☒	The exact sample size (n) for each experimental group/condition, given as a discrete number and unit of measurement
☐	☒	A statement on whether measurements were taken from distinct samples or whether the same sample was measured repeatedly
☐	☒	The statistical test(s) used AND whether they are one- or two-sided<br>*Only common tests should be described solely by name; describe more complex techniques in the Methods section.*
☐	☒	A description of all covariates tested
☐	☒	A description of any assumptions or corrections, such as tests of normality and adjustment for multiple comparisons
☐	☒	A full description of the statistical parameters including central tendency (e.g. means) or other basic estimates (e.g. regression coefficient) AND variation (e.g. standard deviation) or associated estimates of uncertainty (e.g. confidence intervals)
☐	☒	For null hypothesis testing, the test statistic (e.g. F, t, r) with confidence intervals, effect sizes, degrees of freedom and P value noted<br>*Give P values as exact values whenever suitable.*
☒	☐	For Bayesian analysis, information on the choice of priors and Markov chain Monte Carlo settings
☒	☐	For hierarchical and complex designs, identification of the appropriate level for tests and full reporting of outcomes
☐	☒	Estimates of effect sizes (e.g. Cohen's d, Pearson's r), indicating how they were calculated

Our web collection on statistics for biologists contains articles on many of the points above.

Software and code

Policy information about availability of computer code

Data collection	The data in the study represent simple button responses on a standard keyboard and questionnaire data
Data analysis	SPSS was used to analyze the data

For manuscripts utilizing custom algorithms or software that are central to the research but not yet described in published literature, software must be made available to editors and reviewers. We strongly encourage code deposition in a community repository (e.g. GitHub). See the Nature Research guidelines for submitting code & software for further information.

Data

Policy information about availability of data

All manuscripts must include a data availability statement. This statement should provide the following information, where applicable:

- Accession codes, unique identifiers, or web links for publicly available datasets
- A list of figures that have associated raw data
- A description of any restrictions on data availability

The statement provided in the article is as follows" The raw data that support the findings of this study are available from the corresponding author upon request." however we note the supplemental files provide a very comprehensive summary of the data.

Field-specific reporting

Please select the one below that is the best fit for your research. If you are not sure, read the appropriate sections before making your selection.

☒ Life sciences ☐ Behavioural & social sciences ☐ Ecological, evolutionary & environmental sciences

For a reference copy of the document with all sections, see nature.com/documents/nr-reporting-summary-flat.pdf

Life sciences study design

All studies must disclose on these points even when the disclosure is negative.

Sample size	Minimum group sample sizes were based off of similar research studies which had reported significant results using similar designs in this population, e.g. Johnston et al, 2010.
Data exclusions	Six participants (all inpatients) were excluded from the final analyses because they either chose to discontinue early or were discharged before completing the study. No other data were excluded.
Replication	replication studies are not feasible in a clinical study of this type. The initial sample and statistical analysis was designed to determine the extent to which the findings are representative.
Randomization	Not applicable, although the order or tasks was counterbalanced across the sample.
Blinding	blinding was not relevant as this study was not an intervention but represented a standardised assessment protocol for all participants

Reporting for specific materials, systems and methods

We require information from authors about some types of materials, experimental systems and methods used in many studies. Here, indicate whether each material, system or method listed is relevant to your study. If you are not sure if a list item applies to your research, read the appropriate section before selecting a response.

Materials & experimental systems

n/a	Involved in the study	
☒	☐	Antibodies
☒	☐	Eukaryotic cell lines
☒	☐	Palaeontology and archaeology
☒	☐	Animals and other organisms
☐	☒	Human research participants
☒	☐	Clinical data
☒	☐	Dual use research of concern

Methods

n/a	Involved in the study	
☒	☐	ChIP-seq
☒	☐	Flow cytometry
☒	☐	MRI-based neuroimaging

Human research participants

Policy information about studies involving human research participants

Population characteristics	The sample consisted of adult psychiatric inpatients (58% male) who were aged between 19 and 65 (mean age 34) and had a current psychiatric diagnosis. Diagnoses varied but included schizophrenia, schizoaffective disorder, bipolar disorder, drug induced psychosis, first episode psychosis, major depressive disorder, borderline personality disorder, and others. Mean duration of illness was approximately 7.6 years. Healthy controls (18-56 years, mean age 34) were also recruited.
Recruitment	Inpatients were recruited at the Northern Hospital psychiatric inpatient ward. Before inpatients were approached for possible recruitment, the researcher first spoke to their nurse to assess suitability (e.g. current mental state, willingness to engage with others). This means that the sample was biased towards patients who presented as less symptomatic, less paranoid, and more open to socialising with others.
Ethics oversight	Ethical oversight was provided by Melbourne Health and University of Melbourne Human Research Ethics Committees

Note that full information on the approval of the study protocol must also be provided in the manuscript.
