# Supplementary material for: Dynamic face processing impairments are associated with cognitive and positive psychotic symptoms across psychiatric disorders
Source: NPJ Schizophr. 2021 Aug 10;7:36. doi: 10.1038/s41537-021-00166-z (PMC8355323; doi:10.1038/s41537-021-00166-z)
Supplement: Supplementary file 2 — Supplementary Information [file 41537_2021_166_MOESM2_ESM.pdf]

Supplementary Materials

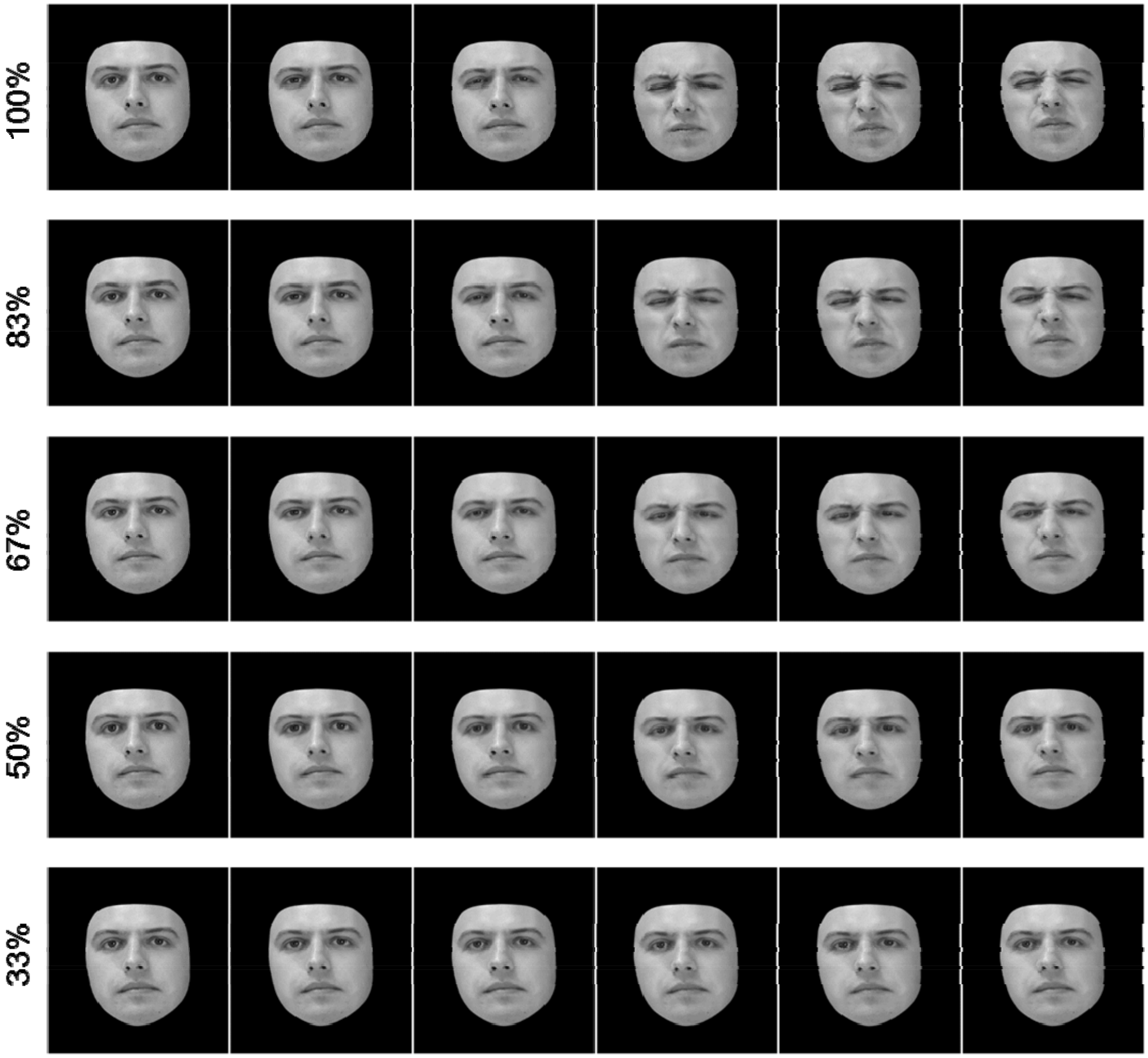

Supplementary Figure 1. The image above shows image sequences from five video stimuli ranging in intensity of emotion. The top video shows the original video of an individual's face changing from neutral to an expression of disgust. This video was then morphed with the neutral face frame (leftmost frame) to reduce the intensity of the final expression (rightmost frame). Emotional intensity ranged from 100% (unmorphed video) to 33% intensity. This method was used to create the stimuli used in Emotion Discrimination and Emotion Labelling.

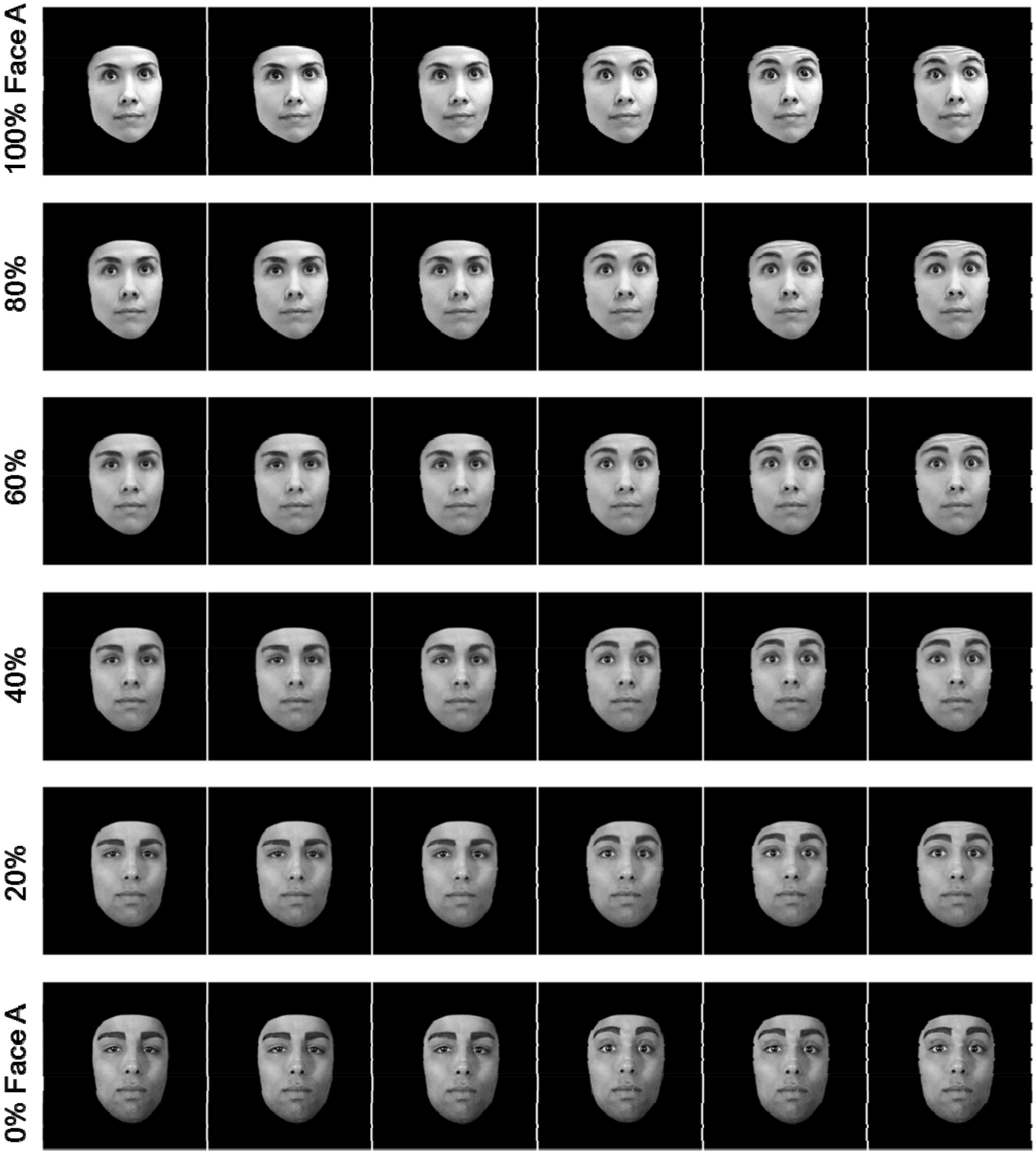

Supplementary Figure 2. The image above shows image sequences from six different videos stimuli. The top and bottom videos show two different individuals making the same motion (eyebrow raise). These videos were then morphed together to create four new videos which vary on a continuum from person A to person B. This method was used to create the stimuli used in Identity Discrimination (individuals of same sex morphed together) and Sex Labelling (individuals of opposite sex morphed together).

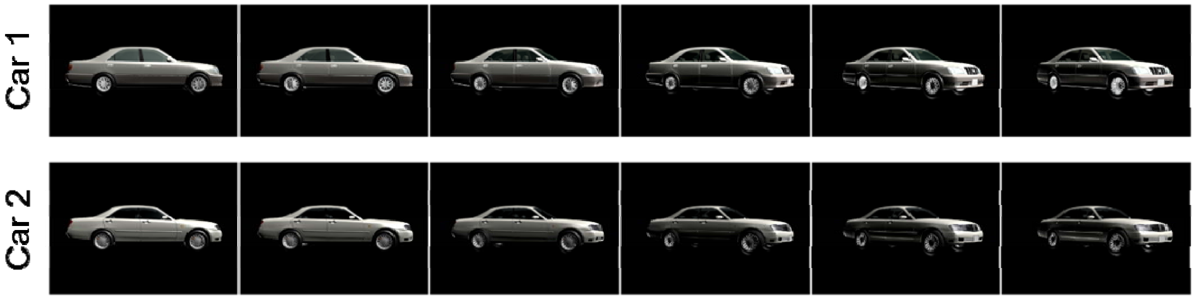

Supplementary Figure 3. Image sequences from two different 3D video stimuli used in the Car Discrimination task. In each video, cars rotate from a side view to a 45 degree view. Car 1 and Car 2 are different models that are similar in appearance.

Supplementary Table 1. Medication status for inpatient groups.

	Neither medication	Antipsychotics only	Benzodiazepines only	Both Antipsychotics and Benzodiazepines
Schizophrenia spectrum	1	28	0	7
Bipolar disorder	0	10	0	5
Other psychotic disorders	5	12	0	0
Non-psychotic disorders	12	2	3	1

Supplementary Note 1: *additional analyses for demographics, PANSS scores, within-group performance, and performance across task morphing levels.*

Demographics

Pearson's chi-square test revealed that the gender makeup of groups did not differ significantly as a function of diagnosis, $X^2(4) = 6.18$, p=.186. One-way ANOVAs were performed with Group as a between-subjects factor and Age, Years of Education, and estimated FSIQ as within-subjects factors. A significant effect was found for Years of Education, $F(4,101)=2.50$, p=.048. Post-hoc t-tests (Bonferroni corrected) revealed a significant difference of 2.11 years between the Control and Schizophrenia group (p=.02). FSIQ estimates were also found to differ between groups, $F(4,93)=2.52$, p=.047. Post-hoc t-tests (Bonferroni corrected) revealed a significant difference of 8.38 points between the Control and Schizophrenia spectrum groups (p=.03). Age did not differ significantly between groups, $F(4,101)$=.75, p=.56.

One-way ANOVAs conducted with the four inpatient groups only revealed no significant group differences in mean duration of illness, $F(3,82)=1.42$, p=.25, mean daily dose of antipsychotics, $F(3,61)=1.68$, p=.18, or daily benzodiazepine dose, $F(2,13)$=.63, p=.55. Medication status for each group is shown in Supplemental Table 1.

PANSS subscales

One-way ANOVAs were run with group as IV (excluding healthy controls), and Positive, Negative, and General Psychopathology scores as DVs. A significant main effect was found for Positive Symptoms, $F(3,82)=18.76$, $p<.001$. Bonferroni corrected post hoc tests revealed, not surprisingly, that the Non-psychosis group had significantly lower Positive symptom scores than all other groups (p=.002 to <.001). The Other group trended towards having significantly lower Positive symptom scores compared with the bipolar group (p=.055). No other group differences approached significance.

Task performance in healthy controls

Supplemental Figure 4 shows the mean performance of healthy controls for the five tasks. To determine whether difficulty varied across the five dynamic tasks in healthy controls, a repeated-measures ANOVA was conducted with accuracy (*d'*) as the dependent variable and task as a within-subjects factor. Mauchly's test showed that the assumption of sphericity was not violated, $X^2(9)=15.64$, p=.08. A significant main effect of task was found, $F(4,76)=7.50$, $p<.001$, η_p^2=.28, indicating that despite attempts to match task demands, difficulty was not uniform across all tasks. Bonferroni-corrected post-hoc tests revealed that performance on Identity discrimination was significantly higher than Sex labelling ($p<.001$, *mean difference* =.92), and Emotion discrimination (p=.004, *mean difference*=.79). No other comparisons were significant (p values=.07-.99). This suggests that the Identity recognition task was slightly less difficult compared to the Sex labelling and Emotion discrimination tasks, however performance across all other tasks was of a comparable level.

Task performance in the schizophrenia group

Mean performance across tasks for the schizophrenia spectrum group are shown in Supplemental Figure 5. As for healthy controls, a repeated-measures ANOVA was conducted with accuracy (*d'*) as the dependent variable and task as a within-subjects factor. According to Mauchly's test, the assumption of sphericity was not violated, $X^2(9)=11.29$, p=.26. A main

effect of task was found, $F(4,132)=53.29$, $p<.001$, $\eta_p^2=.62$. Bonferroni-corrected post-hoc comparisons revealed that performance on the two emotion tasks was not significantly different from one another ($p>.99$) but were both significantly lower than the three remaining tasks (p values>.001, *mean differences* = .76 – 1.58). The identity discrimination task was significantly higher than all other tasks (p values<.001, *mean differences* = .46 – 1.58). Finally, the car discrimination and sex labelling tasks were not significantly different from one another ($p>.99$).

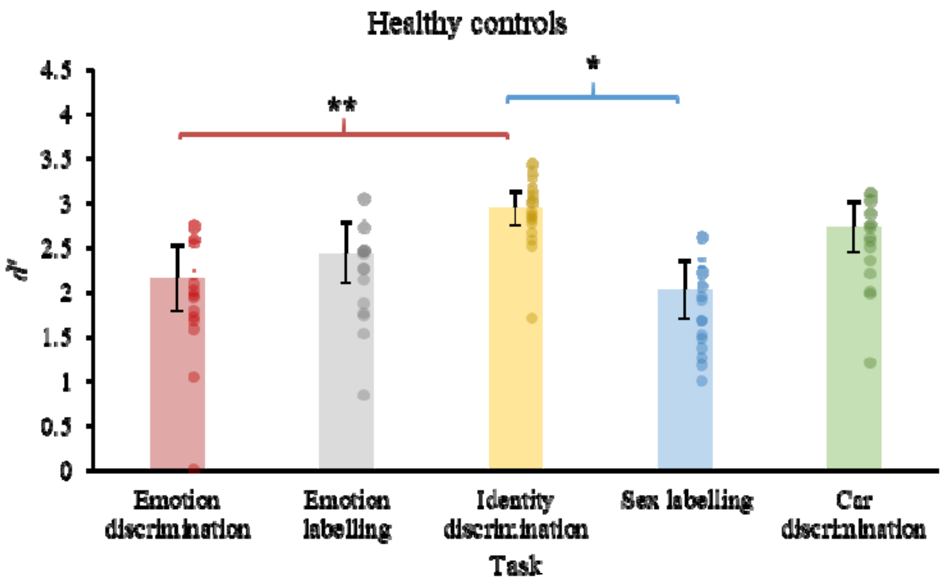

Supplementary Figure 4. Performance (d') of healthy controls across the five dynamic tasks. Error bars indicate 95% confidence intervals. Dots indicate the performance of individual participants. *$p<.01$, **$p<.001$.

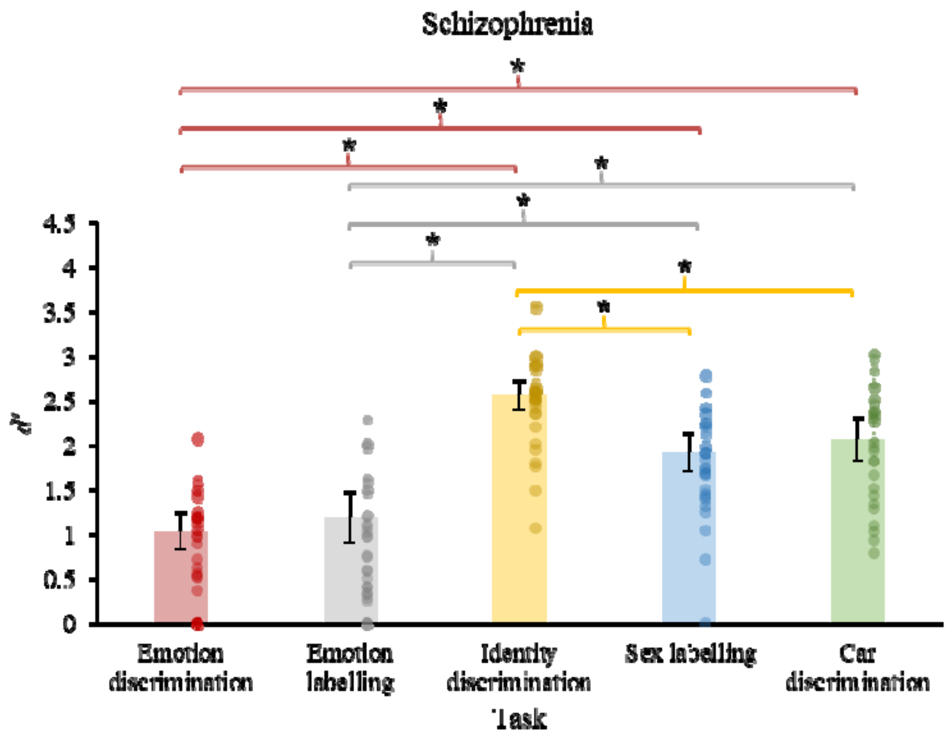

Supplementary Figure 5. Performance (*d'*) of patient with schizophrenia across the five dynamic tasks. Error bars indicate 95% confidence intervals. Dots indicate the performance of individual participants. *p<.001.

Task performance in the bipolar disorder group

Mean performance across tasks for the bipolar group are shown in Supplemental Figure 6. A repeated-measures ANOVA was conducted with accuracy (*d'*) as the dependent variable and task as a within-subjects factor. According to Mauchly's test, the assumption of sphericity was violated therefore the Greenhouse-Geisser correction was used, $X^2(9)=20.74$, $p=.02$. A main effect of task was found, $F(2.18, 30.55)=26.02$, $p<.001$, $\eta_p^2=.65$. Bonferroni-corrected post-hoc comparisons revealed that performance on the two emotion tasks was not significantly different from one another ($p>.99$) but were both significantly lower than the three remaining tasks (p values<.02, *mean differences* = .76 – 1.67). Performance on the identity discrimination task was significantly higher than all other tasks except sex labelling (p values<.01, *mean differences* = .64 – 1.67). Finally, the car discrimination and sex labelling tasks were not significantly different from one another ($p>.99$).

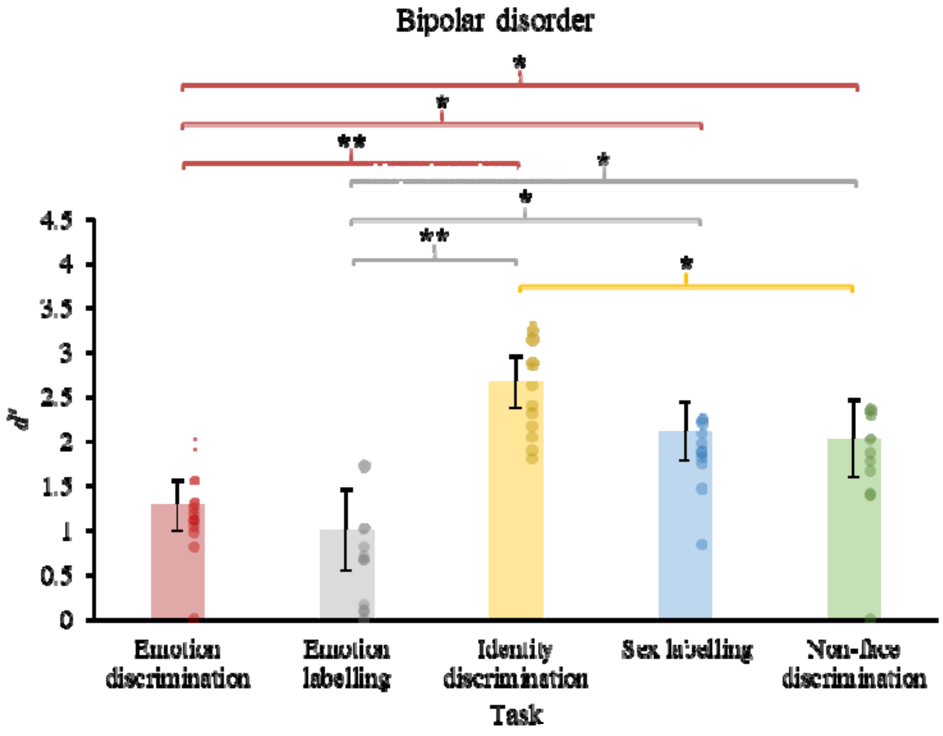

Supplementary Figure 6. Performance (*d'*) of patients with bipolar disorder across the five dynamic tasks. Error bars indicate 95% confidence intervals. Dots indicate the performance of individual participants. *p<.01; **p<.001.

Task performance in the other psychosis group

Mean performance across tasks for the other-psychosis group are shown in Supplemental Figure 7. A repeated-measures ANOVA was conducted with accuracy (*d'*) as the dependent variable and task as a within-subjects factor. According to Mauchly's test, the assumption of sphericity was violated therefore the Greenhouse-Geisser correction was used, $X^2(9)=21.97$, $p=.01$. A main effect of task was found, $F(2.18, 34.80)=9.45$, $p<.001$, $\eta_p^2=.37$. Bonferroni-corrected post-hoc comparisons revealed that performance on the identity discrimination task was significantly higher than the two emotion tasks and the sex labelling task (*p* values <.03, *mean differences* = .66 – 1.08). Performance on the non-face task was also significantly higher than the emotion discrimination task (*p*=.006, *mean difference*=.91). No other comparisons approached significance.

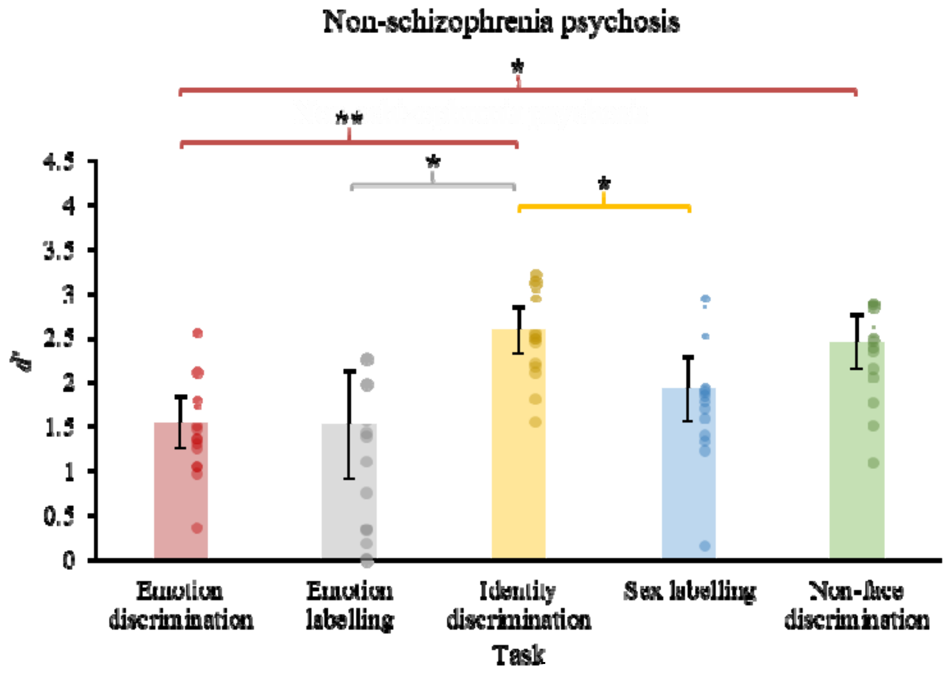

Supplementary Figure 7. Performance (*d'*) of patients with non-schizophrenia psychosis across the five dynamic tasks. Error bars indicate 95% confidence intervals. Dots indicate the performance of individual participants. *$p<.05$; **$p<.001$.

Task performance in the non-psychosis group

Mean performance across tasks for the non-psychosis group are shown in Supplemental Figure 8. A repeated-measures ANOVA was conducted with accuracy (*d'*) as the dependent variable and task as a within-subjects factor. According to Mauchly's test, the assumption of sphericity was not violated, $X^2(9)=14.29$, $p=.11$. A main effect of task was found, $F(4, 68)=13.34$, $p<.001$, $\eta_p^2=.44$. Bonferroni-corrected post-hoc comparisons revealed that performance on the identity discrimination task was significantly higher than the two emotion tasks and the sex labelling task (*p* values<.01, *mean differences* = .87 – 1.01). Performance on the non-face task was also significantly higher than the emotion discrimination task and the sex labelling task (*p* values<.02, *mean difference*=.61-.75). No other comparisons approached significance.

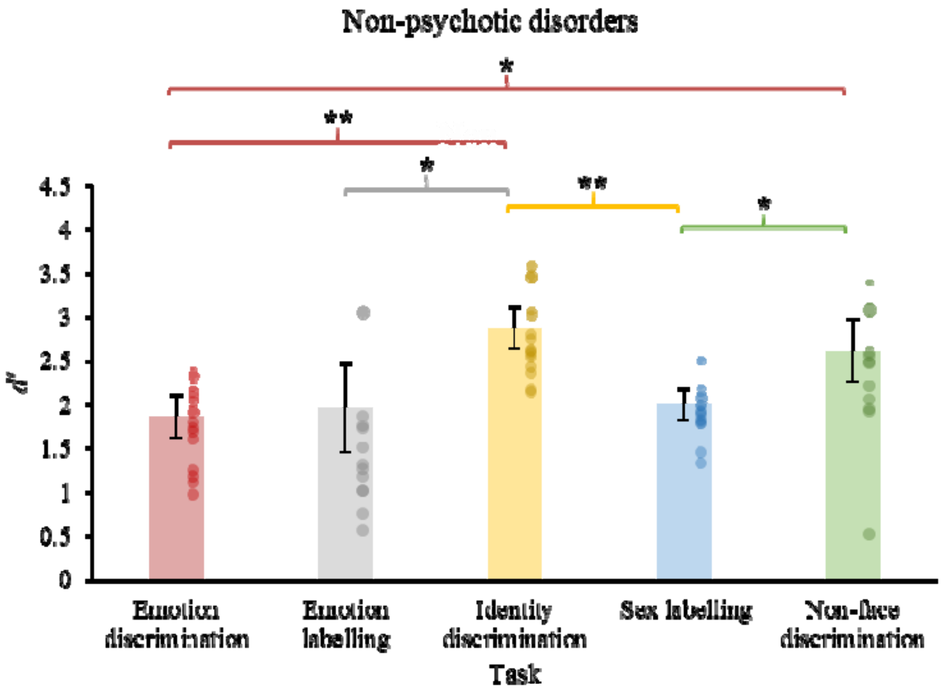

Supplementary Figure 8. Performance (*d'*) of patients with non-psychotic disorders across the five dynamic tasks. Error bars indicate 95% confidence intervals. Dots indicate the performance of individual participants. *p<.05, **p<.001.

Impact of morphing: Varying emotional intensity and facial identity

Emotion Discrimination. Repeated-measures ANOVA was conducted for raw accuracy across the different intensities of expression. The main effect of intensity was significant, $F(1.76, 183.44) = 33.13$, $p = .005$. Contrary to expectations, accuracy was lowest for expressions at 67% intensity compared to all other intensities (p values=.01 to .001), which did not differ significantly from one another. This finding indicates that, for all groups, decreasing the intensity of a moving expression did not significantly affect performance, with one exception. The finding that accuracy was lowest for expressions at 67% intensity likely reflects the increase in visual artefact or "graininess" of the video as a result of the morphing process at this intensity (Supplemental Figure 9A).

Emotion Labelling. A Repeated measures ANOVA on raw accuracy revealed a significant main effect for emotional intensity, $F(3.46, 363.26) = 17.48$, $p < .001$, but not emotion (disgust vs fear). Performance is shown in Supplemental Figure 9B(disgust faces) and 9C (Fear faces). Unlike the Emotion Discrimination task, accuracy for naming emotions increased somewhat with increasing intensity. Performance at 33% intensity was significantly lower than all other levels (p values=.006 - .001), and performance at 50% intensity was lower than 67% intensity ($p = .04$) and 83% intensity ($p = .001$). No other differences in intensity approached significance.

Identity Discrimination. Raw accuracy across different levels of morphing are shown in Supplemental Figure 9D. Repeated-measures ANOVA revealed a main effect of morphing, $F(5,525) = 599.34$, $p < .001$. Accuracy increased significantly with each level of increasing difference (p values = .04 - .001) up to 80% difference, which did not differ significantly from 100% difference ($p > .999$). All groups performed above chance when faces were 60% different, and at or below chance at 40% different.

Sex Labelling. Repeated-measures ANOVA revealed significant main effects of sex, $F(1,104) = 33.13$, $p < .001$ and morphing level, $F(2,208) = 405.05$, $p < .001$. Accuracy was lowest for the 60/40% morphed faces and highest for 100% (un-morphed) faces. Unexpectedly, accuracy was reliably higher for identifying male faces than female faces (Supplemental Figure 9E).

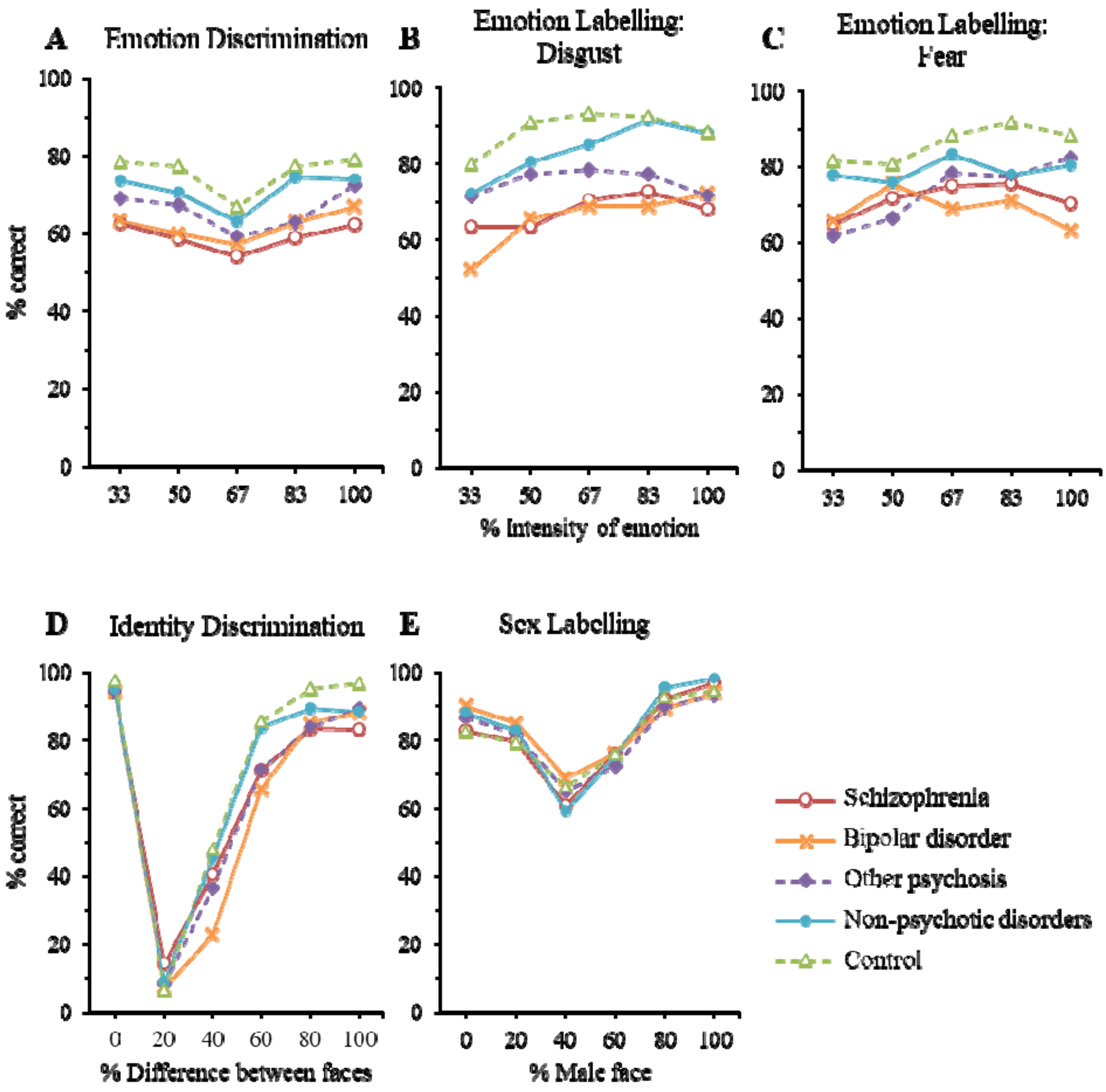

Supplementary Figure 9. Mean accuracy performance for the schizophrenia spectrum, bipolar disorder, non-schizophrenia psychosis (Other psychosis), non-psychotic disorders and control groups across the four morphed face tasks: Emotion Discrimination (A), Emotion Labelling for disgust faces (B), Emotion Labelling for fear faces (C), Identity Discrimination (D), and Sex Labelling (E). For A, B, and C, morphing level is on the y axis, where 100% indicates an unedited expression and 33% indicates an expression morphed 50% with a neutral expression. For D and E, morphing level is shown on the y axis, where 50% indicates an equal morph between Face 1 and Face 2.
